# Metropolitan Hospital Sunday Fund

**Published:** 1904-11-26

**Authors:** 


### METROPOLITAN HOSPITAL SUNDAY FUND.

### REPORT OF THE COUNCIL FOR 1904.

A meeting of the Council of the Metropolitan Hospital Sunday Fund was held on Tuesday, 22nd inst., at the Mansion House, the Lord Mayor (Mr. Alderman John Pound) presiding. There were present the Rev. Prebendary Ridgeway, the Rev. Prebendary Ingram, the Rev. C. H. Grundy, the Rev. R. R. Connell, the Rev. A. A. Harland, the Rev. G. R. Thornton, the Rev. R. S. Hassard, the Rev. E. H. Pearce, the Hon. Sydney Holland, Sir William Church, Sir Henry Burdett, Mr. R. Biddulph Martin, M.P., Mr. C. E. Tritton, M.P., Dr. Glover, Mr. Alfred Willett, and Messrs. J. R. Cooper, Robert Grey, F. H. Norman, and R. de Q. Quincey.

The Secretary, Sir Edmund Hay Currie, presented the following draft report:—

The thirty-second year of collecting this Fund has resulted, under the presidency of the Right Hon. Sir James Thomson Ritchie, Bart., in a total of £63,064 19s. 10d.

The annual general meeting of constituents was held at the Mansion House on Tuesday, December 9th, 1903, when the council for 1904 were elected. At the same meeting Hospital Sunday was fixed for June 12th, and the usual invitations to co-operate were ordered to be sent to all congregations in the metropolitan area.

The collections in the various places of worship resulted in an amount of £47,912 5s. 4d., being, with the exception of last year, when the fund was augmented by the encouragement given by their Majesties' visit to St. Paul's, the largest on record.

The Metropolitan Cathedral of St. Paul's heads the list with £5,490 13s. 0d.; the Rev. Prebendary C. J. Ridgeway, of Christ Church, Lancaster Gate, £1,520; the Rev. Canon Fleming, B.D., of St. Michael's, Chester Square, £1,392; and the Rev. Canon Page Roberts, of St. Peter's, Vere Street, £645. The Rev. Charles Voysey's congregation sent £318, and the Rev. R. J. Campbell's, at the City Temple, £302, after an interval of some years.

The country clergy in the home counties were again invited to join in "Hospital Sunday," and several thousands of residents in the country were also specially asked to assist, but the response was almost *nil*. These country districts furnish approximately 25 per cent. of the in-patients treated in the metropolitan hospitals, and the council feel that there is a strong claim for generous contributions from residents in the country.

Mr. George Herring has again generously added one-fourth to the amount collected in places of worship. This year his gift has been £11,926.

Mr. William Herring and Mr. Charles Morrison gave donations of £1,000 each; Sir Savile Crossley has again divided his contribution of £1,000 between this fund and the King's fund, and "Delta" sent his 26th donation of £200.

A legacy of £500 was paid by the executors of the late Mr. Robert Davidson, and notice of a similar amount has been received under the will of the late Mr. John Cohen.

The editors of newspapers again most kindly and earnestly pleaded the urgent needs of the hospitals, in the week preceding Hospital Sunday.

The working expenses, including rent of offices, for 1904, are £2,931 2s., and are 4·648 per cent. of the gross receipts as compared with 4·166 last year. The average percentage of working expenses since the establishment of the Fund in 1873 is 3·700.

The Council have to record their thanks to the clergy and ministers of all congregations for their continued co-operation, and especially to all those whose pleadings have been followed by an encouraging increase.

The Council have also to thank Mr. Charles Cheston for freely giving his services as honorary solicitor; and Messrs. W. H. Pannell and Co., chartered accountants, for having gratuitously audited this year's accounts.

The Council earnestly appeal to congregations that have not hitherto contributed, to give their aid in the future to this great work of common benevolence.

Some discussion arose on the fourth paragraph of the Council's report relating to the contributions by individual congregations, and eventually it was decided that the first six churches of the Church of England on the list should be mentioned, and that it be left to the General Purposes Committee to select another six whose collections were the highest among the other denominations for inclusion in the paragraph. The report was then adopted.

On the question of filling up vacancies on the Council, it was decided to await the return of Bishop Amigo from Rome before electing him to fill the vacancy caused by the death of Archbishop Bourne. In the place of Mr. W. R. Tidd, deceased, it was decided to request the Corporation of the City of London to nominate a member. In the place of Mr. R. B. D. Acland who was unable to attend the meetings, the late Lord Mayor, Sir James Thomson Ritchie, was elected.

Finally, it was proposed by Prebendary Ridgeway and seconded by Prebendary Ingram to recommend to the annual meeting of the constituents of the Fund in December that the date for Hospital Sunday next year be June 25th. This was adopted, and the meeting closed with a vote of thanks to the Lord Mayor for presiding.

